# Young adults' experiences of seeking online information about diabetes and mental health in the age of social media

**DOI:** 10.1111/hex.12430

**Published:** 2015-12-08

**Authors:** Gillian Fergie, Shona Hilton, Kate Hunt

**Affiliations:** ^1^ MRC/CSO Social and Public Health Sciences UnitUniversity of GlasgowGlasgowUK

**Keywords:** common mental health disorders, diabetes, health information, Internet, social media

## Abstract

**Background:**

The Internet is a primary source of health information for many. Since the widespread adoption of social media, user‐generated health‐related content has proliferated, particularly around long‐term health issues such as diabetes and common mental health disorders (CMHDs).

**Objective:**

To explore perceptions and experiences of engaging with health information online in a sample of young adults familiar with social media environments and variously engaged in consuming user‐generated content.

**Methods:**

Forty semi‐structured interviews were conducted with young adults, aged 18–30, with experience of diabetes or CMHDs. Data were analysed following a thematic networks approach to explore key themes around online information‐seeking and content consumption practices.

**Results:**

Although participants primarily discussed well‐rehearsed approaches to health information‐seeking online, particularly reliance on search engines, their accounts also reflected active engagement with health‐related content on social media sites. Navigating between professionally produced websites and user‐generated content, many of the young adults seemed to appreciate different forms of health knowledge emanating from varied sources. Participants described negotiating health content based on social media practices and features and assessing content heuristically. Some also discussed habitual consumption of content related to their condition as integrated into their everyday social media use.

**Conclusion:**

Technologies such as Facebook, Twitter and YouTube offer opportunities to consume and assess content which users deem relevant and useful. As users and organizations continue to colonize social media platforms, opportunities are increasing for health communication and intervention. However, how such innovations are adopted is dependent on their alignment with users' expectations and consumption practices.

## Background

The Internet has become a key means to access resources relevant to people's health experiences. Although opportunities for users to contribute health‐related content on personal ‘homepages’ were available previously,^1^ the proliferation and widespread adoption of social media since the mid‐2000s have made these opportunities more accessible. The creation of content through social media sites such as Facebook has become commonplace for many users,^2^ allowing for contribution and consumption of accounts of people's lives, including experiences of health and illness. Social media also facilitate peer networks around particular health issues. Such shared links and experiences are said to epitomize the contemporary phenomenon of ‘e‐health’.^3^ Social media also provide increasing opportunities for individuals to engage online to critique health policy and practice.^4^ Like TripAdvisor and Amazon reviews, users' feedback and approval ratings on social media platforms generate useful ‘crowdsourced’ information.

Previous qualitative studies highlighted that the Internet provides resources for facilitating individuals' emergence as ‘lay experts’ or ‘informed patients’,^5^, ^6^, ^7^ drawing on online resources to inform decision making and encounters with health professionals. Exploration of people's everyday engagement with health information online has highlighted users' concerns with about its reliability^5^ and the use of explicit assessment strategies for navigating between resources.^8^, ^9^ However, developments in mobile and social media technologies have precipitated changes in information‐seeking practices. In the UK, the number of users who use multiple and mobile devices to access online content is increasing and reliance on search engines is decreasing, with users choosing to consume information through their online social networks.^10^

Given increasing calls to utilize social media to deliver health information to people with long‐term health conditions, this environment warrants further research. While users' practices for engaging with health information online received attention when Internet access first became widespread, they have yet to be fully re‐examined since the proliferation of social media technologies and pervasion of these into young adults' lives. The main aim of this study therefore was to explore perceptions and experiences of engaging with health information online in young adults familiar with social media environments and variously engaged in consuming user‐generated content.

Previous research on health information‐seeking^11^ and online health information^12^ highlighted the importance of comparing the processes and practices of appropriating information across different health contexts. To focus this research, two contrasting health issues, diabetes and common mental health disorders (CMHDs), were identified as exemplars. Entwistle *et al*.^11^ reflect that using contrasting health issues to explore people's engagement with health information provides a rich source of data because of the range of decision‐making challenges relevant to specific health issues. Given the propensity for health‐related social media use amongst those experiencing long‐term health issues,^13^ people with experience of CMHDs, such as depression, anxiety or stress, and those with diabetes were identified as offering potentially rich insights. Diabetes and CMHDs both also involve some degree of self‐management,^14^ of which information‐seeking online can be seen as a key element.

Yonker *et al.'*s^15^ systematic review of social media use for health purposes amongst adolescents and young adults highlights the multiple opportunities afforded by social media technologies for communication with these groups. The review highlights the high prevalence of mental health‐related content contributed to social media sites by young adults and therefore the multiple opportunities for health professionals' intervention. In relation to diabetes, Jones *et al.'*s^16^ review of social media use amongst young people and young adults with diabetes concluded that the use of social media for seeking diabetes‐related advice was widespread. Indeed, social media technologies seem historically significant in redefining opportunities for communication with individuals experiencing long‐term health issues.

Specifically, therefore, we aimed to explore how the Internet and social media technologies are used by young adults experiencing long‐term health issues and the differences or similarities between people with different health conditions and between men and women.

## Methods

The study was underpinned by an interpretivist approach, focusing on people's understandings of health and illness in the context of their lives.^17^ Ethical approval was granted by the University of Glasgow, College of Social Sciences Research Ethics Committee.

### Data collection

Data were generated through semi‐structured interviews with 40 young adults. A purposive sample of individuals aged 18–30 years, with experience of either diabetes or a CMHD, were invited to participate. Previous studies have successfully used interviews with young adults to explore interpretations of social networking sites.^18^

Recruitment information was posted in a range of locations both offline, in further and higher education institutions, and online, in forum threads and Facebook groups related to diabetes or CMHDs. Study information was also distributed by gatekeepers in organizations for young adults with diabetes or experiencing CMHDs and by participants to peers. Potential participants contacted GF, who then arranged and conducted interviews. Locations included participants' homes (*n* = 12), university buildings (*n* = 11) and cafes (*n* = 17), based on participants' preferences. Table 1 provides an overview of participants' demographic characteristics.

**Table 1:** Participant characteristics

Gender	Health issue	Pseudonym	Age	Education	Length of time since diagnosis or initial experience of health issue
Female	Diabetes	Bronwyn	28	Tertiary	Over 10 years
		Fiona^a^	22	Tertiary	2 years
		Freya	19	Tertiary	9 years
		Ingrid	23	Tertiary	2 years
		Jill	24	Tertiary	Over 10 years
		Leanne	23	Tertiary	2 years
		Mhairi	28	Tertiary	1 year
		Nicola^a^	28	Tertiary	Over 10 years
		Penny	27	Tertiary	Over 10 years
		Poppy	30	Tertiary	4 years
	CMHD	Debbie	30	Tertiary	Over 10 years
		Eleanor	26	Tertiary	8 years
		Fran	25	Tertiary	8 years
		Leah	26	Tertiary	2 years
		Liz	19	Secondary	5 years
		Mia	20	Secondary	6 years
		Sarah^a^	22	Tertiary	7 years
		Simone	25	Tertiary	Over 10 years
		Sylvia	26	Tertiary	Over 10 years
		Tara	21	Tertiary	8 years
Male	Diabetes	Andy	22	Secondary	Over 10 years
		Anthony	28	Tertiary	Over 10 years
		Blake	27	Secondary	Over 10 years
		Byron	18	Tertiary	6 years
		David	29	Secondary	Over 10 years
		Leon	22	Tertiary	2 years
		Max	29	Tertiary	Over 10 years
		Ronan	28	Tertiary	Over 10 years
		Rory	30	Tertiary	Over 10 years
		Tommy	28	Secondary	Over 10 years
	CMHD	Alistair	21	Tertiary	5 years
		Daniel^a^	25	Secondary	1 year
		Euan	28	Tertiary	5 years
		Fraser	28	Tertiary	Over 10 years
		Joe	28	Tertiary	Over 10 years
		Josh	27	Secondary	2 years
		Mike	30	Tertiary	5 years
		Paul	30	Tertiary	9 years
		Peter	19	Secondary	1 year
		Simon	30	Tertiary	1 year

a Denotes pilot participants.

After providing written informed consent, participants were asked about the onset and development of their health issue, then invited to explore examples of online content related to diabetes/CMHDs using a tablet computer. These included YouTube, Facebook and Twitter pages, featuring user‐generated video‐blogs about lived experience of diabetes/CMHDs; images related to diabetes/CMHD self‐management; and extracts of user interaction about diabetes/CMHDs, as well as static websites. Pages were selected to illustrate different forms of content (image, text and video). Participants were also asked to discuss their perceptions and experiences of engaging with online resources, in particular to describe their approaches to information‐seeking, how they negotiated between online resources and how online engagement with health‐related content compared to other online activities. The tablet was available throughout the interview for participants to illustrate key points in their accounts.

Interviews lasted between 40 and 90 min, were digitally recorded, transcribed verbatim and anonymized (pseudonyms used throughout). GF made notes after each interview. All authors reviewed the transcripts at different time points and concluded that enough rich data had been collected after 40 interviews.

### Data analysis

Initial analysis, conducted by GF, was based on the principles of inductive coding, the construction of categories and the continuous comparison of codes described in Grounded Theory.^19^ These codes and categories were then grouped to produce thematic networks, following Attride‐Stirling's^20^ approach. Thematic networks were reviewed by KH and SH and refined based on their reading of the data. Accounts were systematically examined for similarities and differences between men and women and between diabetes and CMHDs.

## Findings

The findings presented here focus on young adults' discussions of their motivations and considerations for accessing online health‐related resources and the processes involved in navigating content. Participants' responses were grounded in their experiences of diabetes/CMHD and reflected their universal familiarity with social media environments.

### Motives and intentions for accessing health‐related content

All participants described accessing online resources to find information about their health condition and presented this as a routine and taken‐for‐granted response to illness. Throughout the interviews, online health information‐seeking was framed as an important part of being a responsible, informed individual. However, there was diversity in how participants discussed their motives and intentions for accessing health‐related content; ‘going online’ was constructed as ‘fact‐finding’, a means of accessing other people's experiences and an activity that was time and context dependent. All of these constructions of online information‐seeking involved the consumption of both professionally produced and user‐generated content, but emphasis was often placed on the value of one or the other dependent on how the activity was framed. ‘Fact‐finding’ was generally associated with professionally produced content and accessing others' experiences with user‐generated content. However, participants described moving between these different types of content as an integral part of information‐seeking online. Generally, there were few differences in discussions of motives and intentions according to the participants' specific health conditions, and there were no notable differences by gender.

#### Fact‐finding

Most commonly, participants discussed accessing health‐related content online as a means of sourcing ‘factual’ information, ranging from basic descriptions of symptoms to specific treatment‐related queries. For instance Mia said: ‘I started to feel like, “well, maybe it is depression,” and I did, Google it – the usual stuff, and I thought, “yeah, this must be it”’ (Mia, CMHD, 20). Mia positioned her investigations via Google as central in her account of realizing that she might be experiencing depression. She referred to searching online as ‘the usual stuff’, identifying it as a self‐evident practice in developing an understanding of her experience, including identifying symptoms. These types of references to the Internet and online searching were common.

Some also associated online information‐seeking with a concern for finding ‘facts’ from reputable sources. For instance, Poppy stated:

I know that if I want some factual advice, […] if I want to know say the NICE [UK National Institute for Clinical Excellence] guidelines, stuff like this, I know where to get them and that's really useful. […] I've Googled ‘contraception’ once which was a good job because my doctor was talking rubbish (Poppy, diabetes, 30)

Poppy described her search for resources related to the advice she was given by health professionals and seemed to rate ‘factual’ online resources, such as NICE guidelines, as more reliable than her doctor's advice. She emphasized the importance of online information, as opposed to information which has been mediated, interpreted and relayed by another source, such as a health professional. Although not all participants expressed a preference for online resources over health professionals' advice, for most going online was crucial for scoping information sources. Indeed, health professionals' advice was not discussed in these terms; their advice was presented as a singular source of information rather than a gateway to a range of sources of knowledge.

#### Accessing others' accounts

While ‘fact‐finding’ was the dominant explanation of participants' motives for online activity, some discussed using the Internet to seek accounts from other people with similar health conditions. For example, Nicola commented:

the organisations [diabetes charities], they're not the people who are actually dealing with it [diabetes] on a day‐to‐day basis, […] the burden of having it […] I think these [diabetes Facebook pages], they're good […] for just knowing that there are other people that have the same [condition] (Nicola, diabetes, 28)

Some participants also sought out content that was consistent with their particular experience:

When I've been feeling down I've Googled ‘other people feeling down’, […] just to see what they're dealing with, […] I don't want to know their problem, I just like to know that you're not the only person that feels that way. […] It's nice to know that people understand, you know, how it can beat you (Alistair, CMHD, 21)

Despite having been uninterested in the specific circumstances of other people's mental health problems, Alistair sought experience‐based content to help him feel less isolated and validate his experience. For some young adults, online information‐seeking was contingent on other users contributing content about their experiences. Searching and consuming user‐generated content seemed to relieve feelings of isolation and provide emotional support.

#### Combining ‘fact‐finding’ and accessing others' accounts

Although most participants distinguished bet‐ween ‘fact‐finding’ and accessing others' accounts as different forms of information‐seeking, for some these activities appeared less distinct. For example, when asked what resources he had used to learn about diabetes, Max commented:

Everything from the internet, […] I never really understood what the theory was – I just used to take it [insulin] until I learned why I was doing it, and what the reasons were. I think that's how I built that understanding of what the basal insulin does. And, I've learned about that from people who use the pump [device for administering insulin] (Max, diabetes, 29)

This extract illustrates the diversity of resources drawn on when seeking information online. Max did not clearly distinguish between professionally produced and user‐generated content, but highlighted experience‐based content as an important resource for informing his self‐management practices. For some participants, the Internet was constructed as a dynamic resource, a space for active negotiation of diverse complementary resources. By combining the processes of ‘fact‐finding’ and accessing others' experiences online, some participants described building their knowledge base for self‐management practices.

#### Timing and context: critical junctures, on‐going concerns and everyday engagement

When asked about accessing health‐related content, many participants discussed being motivated to access content in response to a specific issue, concern or query for which they needed timely information. Perhaps the most striking instance was described by Joe:

[…] the last time I was really badly suicidal and was self‐harming, there was a web page that, when I felt really suicidal and I felt like I was going to actually do it, I would open, I had it as a shortcut on my desktop and I would open it and the time it took me to read it would quite often let the suicidal feeling subside (Joe, CMHD, 28)

Joe's account highlights the time‐dependent nature of accessing content. For many participants, during times of crisis, when offline supportive resources were either unavailable or unhelpful, specific health‐related content seems to have provided timely and necessary assistance.

Participants also provided examples of instances when they had drawn on online resources in less critical circumstances in response to general concerns about their health or self‐management, often related to day‐to‐day challenges. For instance, some diabetic participants discussed online content as a convenient resource to inform self‐management practices related to exercise. Similarly, Liz who had experienced low mood and anorexia for the past 5 years discussed her experience of engaging with online content for an on‐going concern:

There's a website called Calorie Count […] it's got different forums, and it's got one for gaining weight, […] and there's, like ‘at school, how do you manage to eat this amount of calories?’, or whatever, cos obviously everybody else is going through the same sort of thing, and they'll be like, ‘oh, I eat a lot of peanut butter’ […] I think that's helpful, because, I've got loads of people around me, but nobody else is gaining weight (Liz, CMHD, 19)

For most participants, therefore, online information seemed to be drawn on in response to time‐sensitive issues to inform either immediate decision‐making or on‐going concerns about self‐management practices. In contrast, a few participants discussed accessing online information as a habitual practice embedded in their everyday activities. These were mainly individuals who regularly engaged with health‐related content online. For instance, Eleanor said:

Vitamin C for example, […] was one of the things I was reading up on and […] Wikipedia says it's not founded but there is studies that suggest…, but I think well I'll give it a try because if I go on to forums other people have great success […] I think that the best way to keep up with research is to cover all angles, you go through official angles, and then you'll maybe have a wee Google […], go on to the health bits on […] news websites to see if there's anything that catches my eye […], there's always a link to something or other (Eleanor, CMHD, 26)

Eleanor, as one of the most active users of online resources, explained how her consumption of online content and her self‐management practices were closely related. Unlike many participants who accessed content responsively, consumption of diverse online resources seemed to have become embedded in her day‐to‐day health experiences.

### Navigating health‐related content online

Participants also shared their experiences of navigating online resources and the processes of locating and assessing content. Again, there were few differences in how participants described these processes according to their particular health issue (diabetes/CMHD) or gender. Rather, practices for negotiating health‐related content seemed connected to their day‐to‐day online activity.

#### Locating relevant content

Participants spoke at length about how they located relevant content. Most commonly, they discussed the necessity of searching and, in particular, using Google to locate useful online content:

I will go to Google, […] So I say [type] ‘diabetes’, and […] more often than not I'd go to the first one [result] just the top one, go on to it, and if that doesn't answer what I'm trying to find, I'll go to the next one (Fiona, diabetes, 22)

If I was feeling absolutely desperately unhappy I'd maybe type in ‘what to do when you're feeling desperately unhappy’. Like, do literally the most basic thing in the world, and I'd probably read the first few pages, just glance at them (Mia, CMHD, 20)

Like most participants, Mia and Fiona discussed relying on search engines; they rarely described visiting sites directly via URL. Many, like Fiona, described searching for specific key words, while others used longer search queries like Mia's. Individuals' searching practices impacted the resources they accessed, because most mentioned only viewing the first few links returned by the search engine. Throughout the interviews, the importance of searches was stressed and ‘Googling’ seems to remain important to active navigation of online resources.

However, participants' accounts of navigating resources were not solely focused on searching. For some, locating relevant content involved more than ‘Googling’:

it's a bit more informal isn't it or, you know, like if you're on Facebook, […] you go off on tangents, […] you skip from one thing to the other. […] You might just kinda think ‘yeah, well that's quite interesting, I'll read a bit more into it’, and then yeah, it might lead onto looking at the website (Simon, CMHD, 30)

Simon described an approach to navigating content facilitated by following links on social media sites, in contrast to discussions of targeted searching. Implicit in Simon's account is a more social aspect of navigation. Due to the nature of Facebook, the content to catch his attention comes directly or indirectly from his online social networks. Max mentioned this more explicitly:

[Administrator of diabetes Facebook group] quite often posts links, yeah, the latest stuff that's going on, and anything from the news […] that quite often leads me to go and have a look into these things […] and I've done further research, and found out more about a product, or something that's happening (Max, diabetes, 29)

This less focused process of following links and recommendations was presented as an alternative means of finding and appropriating health‐related content. Although some discussed utilizing both approaches successfully, the less focused approach was emphasized most by participants who engaged frequently online, as everyday consumers of health‐related content.

#### Assessing online resources: determining credibility and reliability

Consistently, participants' discussions of accessing online resources were permeated with references to concerns about misinformation. Comments such as ‘obviously any online activity should be taken wi’ a pinch o' salt' (David, diabetes, 29) and ‘but, again, you don't really want to trust anything over the Internet’ (Liz, CMHD, 19) were common. The emphasis conveyed through language, such as ‘obviously’ and ‘again’, suggests shared assumptions around conscientious consumption of online health information. Despite comments around the limitations of online content – particularly that contributed by other users on social media sites, participants generally presented their own online activities as successful. They provided examples of a range of assessment strategies, including comparing multiple sources, evaluating the purpose of content and assessing the visual impact of content.

The practice of comparing multiple information sources was mentioned by most participants as an important means of assessing the credibility and reliability of health‐related content.

Basically with all the information that I see online […], everything that pops up, somebody says: ‘This is quite a good move’, well the first thing I'm going to do is go on Wikipedia, is go to the forum, is go on Google, like PubMed just to see, ‘is that backed up by the information that I can find elsewhere?’. So all of these [individual resources] are only ever used as the starting point (Anthony, diabetes, 28)

Anthony's account reflects the complexity and multifaceted nature of negotiating health‐related content online; different sources with different types of information, delivered through different media, are brought together by users as they browse. Navigating between diverse resources, and comparing content, seemed an important part of determining the reliability of information. As with much health‐related online activity, this strategy seems linked to day‐to‐day online practices, with regular movement between user‐generated content and professionally produced resources commonplace in accounts.

Participants also reflected on specific strategies for assessing the reliability and relevance of individual resources. For many, making distinctions about the purpose of content was crucial in determining the reliability of information and its relevance. For example, when asked about how she determined whether information encountered was reliable, Nicola said:

it depends […], where you get it from, so if it was an official sort‐of diabetes type website, then I would assume it is reliable, whereas a Facebook page, I wouldn't necessarily assume it […] I suppose it depends [on] the kind of information you are wanting to get […] If it was like a comment on a Facebook page […], if you did more reading and you found that lots of people had the same opinion or same experience then I would be more inclined to believe it than some random person saying something (Nicola, diabetes, 28)

During her interview, Nicola distinguished between professionally produced content (‘official sort‐of diabetes type website’) and social media content (‘Facebook page’). She suggested that the value of these resources lies in her particular needs at the time. While participants often reported distrusting user‐generated content as ‘fact’, they described judging it by different standards, valuing it as a source of opinion. For some participants, like Nicola, ‘crowd‐sourcing’ these opinions was sometimes useful.

As part of the process of evaluating the credibility and reliability of online content, participants mentioned identifying hallmarks such as logos, recognizable URLs and ‘about’ sections. However, most commonly, they articulated the importance of the visual impact of websites. For example, Leon commented:

I think seeing a website that's well put together…, it kind of… not builds trust, but you tend to think ‘Oh, this looks proper.’ […] Yeah, I'll stay here and have a look at this (Leon, diabetes, 22)

Participants emphasized that during rapid online navigation, websites must appear ‘proper’ (well structured and well designed) and salient (containing images and language that seemed relevant to their own experiences):

the homepage needs to have some sort of interesting picture, without someone putting their head in their hands, because that is what all the homepages have […] that's not uplifting! Seeing that is just like, ‘Oh dear. Great. If you're comparing me to this person, my head isn't in my hands,’ […] it puts you off (Mia, CMHD, 20)

Have you heard of ‘Young, Fun and Type 1’? It's a blogger […] it's really, really good. […But] there are pages on Facebook, which when I first was diagnosed when I was [searching Facebook for] ‘diabetes’, there's like pages that are ‘I hate my diabetes’ […] so, yeah, avoided them (Mhairi, diabetes, 28)

These extracts highlight how images and the tone of content can inform initial impressions of relevance and influence users' decision making about content consumption. Processes of evaluation seemed not only embedded in everyday Internet use, but also influenced by conceptions of self and health experiences.

## Discussion and conclusion

This study highlights how the ‘re‐ordering’ of boundaries precipitated by social media technologies^21^ is enacted in the context of young adults' experiences of information‐seeking in relation to diabetes and CMHDs. The findings reflect how boundaries between ‘lay’ and professionally produced content are re‐drawn or made permeable through social media use, exemplifying Nettleton's^22^ concept of ‘e‐scaped’ medicine: ‘Medical knowledge is no longer exclusive to the medical school and the medical text; it has ‘escaped’ into the networks of contemporary infoscapes where it can be accessed, assessed and reappropriated (p. 179)’. Participants in this study discussed actively and effortlessly negotiating between evidence‐informed, professionally produced content and user‐generated content. Many utilized social media to inform self‐management strategies, drawing on others' experiences or niche content brought together by issue‐specific online communities.

Several qualitative studies have discussed the central role of online resources in facilitating individuals' emergence as ‘lay experts’ or ‘informed patients’.^5^, ^6^, ^7^ In this study, throughout accounts, ‘going online’ was taken for granted as the primary means of accessing health‐related information. Previous research has indicated that older adults are less likely to trust online health information^23^ and highlighted barriers to online health information‐seeking amongst adults, such as concerted avoidance of self‐monitoring, varied information literacy levels and wariness of affecting doctor–patient relationships.^24^, ^25^ In this study, however, few accounts reflected any reluctance to access health information online. Rather, ‘going online’ was described as a key means of engaging with health information, reflective of the young adults’ day‐to‐day experience of online activity generally. This aligns with Edwards *et al*.'s^26^ suggestion that willingness to use the Internet for health interventions reflects previous exposure to, and confidence in, online technologies.

Accounts of *why* participants were drawn to online resources varied. For most ‘fact‐finding’ was prioritized, consistent with conceptions of the ‘informed patient’, while for some going online was a means of gaining insights into others' experiences. The latter rationale resonates with the findings from both mental health and diabetes literature which suggests that social media facilitate productive and supportive interactions.^16^, ^27^, ^28^ Indeed, participants seemed drawn to online resources whenever offline support was unavailable or unhelpful. Furthermore, these two activities, ‘fact‐finding’ and accessing others' experiences, were not always presented as distinct; rather, some participants described them as part of the same scoping process.

Previous research has highlighted the role of Google as the primary gatekeeper to relevant health information.^29^, ^30^ Mager^29^ analysed data collected in 2006–2007 from 40 individuals tasked with finding information online about a particular chronic disease assigned to them by the researchers. The importance of Google was evident, as it ‘actively mediated and shaped what information users ended up with, and how they interacted with and evaluated bits and pieces of information they found on various websites’ (p. 15). While our findings align to a degree, alternative practices for locating content related to diabetes or CMHDs, through following links or recommendations from social media contacts, were also highlighted.

Less targeted information‐seeking online was emphasized particularly by participants who were not seeking health‐related content responsively but as everyday consumers. Alongside using search engines, accessing relevant content through social media was described, in accordance with Dutton *et al.'*s^10^ suggestions about changing online information‐seeking practices more broadly. They suggest that declining search engine use is related to users increasingly relying on links and recommendations from within their online social networks, and use of embedded search functions on preferred social media sites, such as Wikipedia and YouTube. As users' practices develop and information‐seeking becomes increasingly linked to social media and social networking practices, users encounter a range of influences, from both identifiable online relationships and marketing based on their browsing habits. Just as Mager^29^, ^30^ emphasized that search engines are not passive technologies, delivering content without filtration or prioritization, social media are similarly not neutral, but impacted by a range of explicit and tacit influences, including peer networks and commercial interests. With users increasingly employing social media to locate and consume relevant health‐related content, the impact of these influences requires further research. Furthermore, the fluidity with which participants described moving between professionally produced and user‐generated content, as well as between static websites and social media sites and forums, highlights the importance of thinking about content consumption as a process, not as a singular activity. Indeed, it seems that users do not clearly distinguish between resources; often, user‐generated contributions to social media platforms involve the sharing of content professionally produced in another context. Further exploration of how interpretations of content shift with context could also inform how best to utilize social media for health communication.

The findings of the study also suggest that on the whole, participants' perceptions and experiences differed according to neither their health issue nor their gender, but rather according to their approaches to wider, everyday Internet use. Accounts were permeated with references to reliability and trustworthiness, similar to previous studies of everyday engagement with health information online.^5^, ^8^ Furthermore, accounts of navigating content suggested explicit assessment strategies, similar to previous research. This has been noted previously in relation to general online health information,^9^ but not fully explored in the context of increasing health‐related content on mainstream social media sites. Our findings suggest the use of tailored approaches to assessing the varying types of content, be it clinical information or accounts of others' experiences. The fast‐paced nature of navigating health content online also seems to result in rapid judgements based on initial impressions and heuristic assessment. The strategies reported here, for assessing health‐related content, aligned with Metzger *et al*.'s^31^ ‘consistency’ heuristic, involving comparing content to ensure consistent information; ‘expectancy violation’ heuristic, involving identifying content as context appropriate; and ‘endorsement’ heuristic, involving recognizing recommendations. These approaches are further complemented by social media practices, such as the ‘like’ function which makes endorsement visible, and the standard structure of Facebook and Twitter pages which facilitates the assessment of ‘expectancy violations’. For many of the young adults, it seems that the consumption of content is largely based on these heuristic assessment strategies, alongside personal tastes or perspectives, grounded in their everyday online consumption of news or entertainment‐based content.

This study explored young adults' perceptions and experiences of engaging with content related to either diabetes or CMHDs. The findings provide insights into the diverse ways that social media can impact the processes and practices of online information‐seeking. In particular, participants' accounts suggested that social media practices can inform both how health‐related content is accessed and consumed and how it is assessed and appropriated. However, the study had some limitations. Most participants had received/were currently studying for degree‐level qualifications, and most were employed or in full‐time education. All had daily access to online technologies at home, and most had smartphones. This limits the extent to which our findings can contribute to debates about ‘digital divides’ based on socioeconomic status and the impact of social position on information‐seeking practices.

Social media technologies offer opportunities to consume and assess content which users deem relevant and reliable. As users and organizations colonize these online spaces, consumption practices will continue to evolve. In particular, accessing niche content through social media platforms seems important for users with long‐term health issues who seek specific information or maintain interests beyond evidence‐informed information and dominant medical perspectives. For organizations attempting to disseminate information online to support self‐management of either diabetes or CMHDs, it is crucial to maintain awareness of how users draw on dynamic social media content to create opportunities for contributing to everyday discussions online. Organizations providing health information for young adults should recognize that while ‘going online’ is perhaps the primary means for accessing health information, the landscape of online health information is diverse. Users navigate resources based on their specific needs, preferences and everyday practices, and information providers must respond by creating content which is responsive to this intertextual and multimedia environment. Users appear less likely than ever to read entire websites or webpages, instead drawing together text, image and video content from various sources to inform their understanding and self‐management practices.

## Funding

G.F. and S.H. were funded by the UK Medical Research Council as part of the Understandings and Uses of Public Health Research Programme (MC_UU_ 12017/6) at the MRC/CSO Social and Public Health Sciences Unit, University of Glasgow. K.H. was funded by UK Medical Research Council as part of the Gender and Health Programme (MC_UU_12017/3) at the MRC/CSO Social and Public Health Sciences Unit, University of Glasgow. The Medical Research Council had no involvement in the design of the research.

## Conflicts of interest

The authors declared no conflict of interest with respect to the research, authorship and/or publication of this work.
